# Ginsenoside Rg1 Ameliorates Cigarette Smoke-Induced Airway Fibrosis by Suppressing the TGF-*β*1/Smad Pathway In Vivo and In Vitro

**DOI:** 10.1155/2017/6510198

**Published:** 2017-03-21

**Authors:** Sibin Guan, Qian Liu, Fengfeng Han, Wen Gu, Lin Song, Yue Zhang, Xuejun Guo, Weiguo Xu

**Affiliations:** Department of Respiratory Medicine, Xinhua Hospital, Shanghai Jiao Tong University, School of Medicine, 1665 Kong Jiang Road, Shanghai 200092, China

## Abstract

Small airway fibrosis is a key pathological process accompanying chronic obstructive pulmonary disease (COPD) and includes fibroblast/myofibroblast transdifferentiation and excessive extracellular matrix deposition. Ginsenoside Rg1, one of the main active ingredients of* Panax ginseng*, has been shown to exert an antifibrotic effect in many tissues. However, little is known about the underlying mechanism and whether ginsenoside Rg1 can exert an effect on small airway fibrosis. We investigated the anti-small airway fibrosis effects of ginsenoside Rg1 in human embryonic lung fibroblasts and in COPD rats. We found that ginsenoside Rg1 effectively reduced the degree of pulmonary fibrosis, decreased the expression of *α*-smooth muscle actin, collagen I, and matrix metalloproteinase 9, and maintained the ratio of matrix metalloproteinase 9 to tissue inhibitor of metalloproteinase 1. Importantly, ginsenoside Rg1 significantly attenuated cigarette smoke extract-induced upregulation of transforming growth factor *β*1, TGF-*β* receptor I, phospho-Smad2, and phospho-Smad3. In addition, ginsenoside Rg1 mimicked the effect of SB525334, a TGF-*β* receptor I-Smad2/3 inhibitor. Collectively, these results suggest that ginsenoside Rg1 may suppress cigarette smoke-induced airway fibrosis in pulmonary fibroblasts and COPD rats by inhibiting the TGF-*β*1/Smad signaling pathway.

## 1. Introduction

Chronic obstructive pulmonary disease (COPD) is a worldwide public health problem characterized by progressive airflow limitation [1]. Small airway fibrosis has been reported to be a key pathogenic component of COPD [2] and contributes to pulmonary remodeling. Presently, no effective treatment for airway fibrosis has been developed, and current pharmacological therapies can only suppress symptoms and disease progression to some extent.

Small airway fibrosis is a complex process, which includes increased transdifferentiation of pulmonary fibroblasts into myofibroblasts, characterized by upregulation of *α*-smooth muscle actin (*α*-SMA). In response to inflammatory stimulation, the persistent activated pulmonary fibroblasts/myofibroblasts secrete various cytokines and growth factors, leading to a protease/antiprotease imbalance and abnormal production of extracellular matrix (ECM). Cigarette smoke (CS) is a major risk factor for the development of COPD [3] and has been shown to cause chronic airway inflammation and induce lung fibroblast-to-myofibroblast transition [4, 5]. Thus, inhibiting the activation of pulmonary fibroblasts could be an attractive therapeutic target in treating small airway fibrosis.

*Panax ginseng*, a traditional herbal medicine, exerts pharmacological effects on inflammation [6], carcinoma [7], and aging [8]. In Chinese medicine,* P. ginseng* has long been used to treat respiratory disease. Ginsenoside Rg1 is one of the primary active compounds in* P. ginseng *and has been shown to inhibit inflammation and oxidative stress [9, 10]. Zhang et al. demonstrated that ginsenoside Rg1 could alleviate myocardial fibrosis induced by transverse aortic constriction [11]. Xie et al. found that ginsenoside Rg1 significantly attenuated the development of renal interstitial fibrosis in a rat unilateral ureteral obstruction model, perhaps through a reduction of transforming growth factor *β*1 (TGF-*β*1) [12, 13]. In addition, Li et al. showed that ginsenoside Rg1 suppressed CCl_4_-induced hepatic fibrosis through the Nrf2 pathway [14]. However, the antifibrotic molecular mechanism of ginsenoside Rg1 in small airway fibrosis associated with COPD remains poorly understood.

The current study was performed to determine whether ginsenoside Rg1 could suppress airway fibrosis in COPD rats and in pulmonary fibroblasts induced by cigarette smoke extract (CSE), and to identify the molecular signaling pathways involved, thus evaluating whether ginsenoside Rg1 could act as an attractive remedy for COPD.

## 2. Materials and Methods

### 2.1. Animal Model and Experiment Design

All animal experimental protocols were approved by the Ethics Committee of Xinhua Hospital affiliated with the Shanghai Jiao Tong University School of Medicine (number XHEC-F-2016-024). Eight-week-old male Sprague-Dawley rats were purchased from SLAC Laboratory Animal Co. Ltd. (Shanghai, China). Ginsenoside Rg1 (purity >98%, MW: 801.01) was purchased from Urchem Sinopharm Chemical Reagent Co., Ltd. (Shanghai, China). All rats were randomly assigned to a Sham group, Sham + Rg1 (20 mg/kg/d) group, COPD group, and COPD + Rg1 (20 mg/kg/d) group (*n* = 8 per group). COPD was induced by exposure to cigarette smoke (Da Qian Men cigarettes, Shanghai Tobacco Company, Shanghai, China) according to a previously described method [15]. Ginsenoside Rg1, dissolved in sterile distilled water, was intragastrically administered 30 min before CS exposure. The Sham group and COPD group were intragastrically administered sterile distilled water (2 mL per animal). Twelve weeks later, all rats were sacrificed under anesthesia to collect samples for subsequent experiments.

### 2.2. Cell Culture

Human embryonic lung fibroblasts (MRC5 fibroblasts), obtained from the Cell Bank of the China Science Academy (Shanghai, China), were cultured in minimum essential medium (MEM, Gibco, Carlsbad, CA, USA) containing 10% fetal bovine serum (FBS, Gibco) at 37°C in a 5% CO_2_ atmosphere. After cells reached 70–80% confluence, the medium was replaced by serum-free MEM the day before pretreatment with ginsenoside Rg1 or selective inhibitor of TGF-*β* receptor I (SB525334, Selleck Chemicals, Houston, TX, USA) and stimulation by CSE. CSE was prepared using a modified method described previously [15]. MRC5 fibroblasts were divided into seven groups: (1) Sham, (2) Sham + Rg1 (40 *μ*M), (3) Sham + SB525334 (3 *μ*M), (4) CSE (10%), (5) CSE (10%) + Rg1 (40 *μ*M), (6) CSE (10%) + SB525334 (3 *μ*M), and (7) CSE (10%) + Rg1 (40 *μ*M) + SB525334 (3 *μ*M). After 48 h of incubation, cell supernatants were collected for ELISA analysis and the cells were harvested for western blot analysis.

### 2.3. Cell Viability Assay

CSE and ginsenoside Rg1 cytotoxicities were evaluated using a Cell Counting Kit-8 (CCK-8) assay (Dojindo, Kumamoto, Japan). Ginsenoside Rg1 (20 mg) was dissolved in 20 mL of MEM sterilized through a 0.22 *μ*m pore filter and then diluted to the required concentration. Briefly, MRC5 fibroblasts (3 × 10^3^ cells/well) were seeded into 96-well plates in 100 *μ*L of serum-free medium for 6 h at 37°C, followed by incubation with CSE (5%, 10%, 15%, and 20%) or ginsenoside Rg1 (5 *μ*M, 10 *μ*M, 20 *μ*M, 40 *μ*M, 80 *μ*M, and 160 *μ*M). After 12, 24, 48, or 72 h, 10 *μ*L of CCK-8 was added to each well, and the cells were returned to the 37°C incubator for an additional 2 h. Absorbance was measured at 450 nm using an ELISA reader (MQX200R, BioTek, Winooski, VT, USA).

### 2.4. Airway Histological Analysis and Immunohistochemistry

The right upper lobe lung was removed and fixed in 4% paraformaldehyde. After embedding in paraffin, the samples were cut into 4 *μ*m thick sections. Some sections were stained with hematoxylin and eosin, and others were subjected to Masson trichrome staining for airway fibrosis analysis or immunohistochemical staining for *α*-SMA detection. Sections were incubated with an anti-*α*-SMA antibody (Abcam, Cambridge, UK) at 4°C overnight and then incubated with anti-mouse horseradish peroxidase- (HRP-) conjugated secondary antibody at 37°C for 30 min. All slides were imaged using an Olympus microscope (Tokyo, Japan). The mean linear intercept (MLI) and mean alveolar number (MAN) were calculated to assess the degree of lung emphysema.

### 2.5. Immunofluorescence Analysis

MRC5 fibroblasts were grown on 24 mm glass coverslips and pretreated with ginsenoside Rg1 (40 *μ*M) for 1 h and then stimulated with 10% CSE for 48 h. The cells were fixed with 4% neutral formaldehyde solution and permeabilized with 0.1% Triton X-100. After blocking in 1% bovine serum albumin (BSA) for 30 min at 37°C, the slides were incubated with a primary antibody against *α*-SMA (Abcam) overnight at 4°C, followed by Cy3-conjugated secondary antibody for 1 h. Finally, 4′6-diamidino-2-phenylindole dihydrochloride (DAPI) was added for cell nuclei detection. Fluorescence staining was visualized using a fluorescent microscope (Olympus).

### 2.6. Cytokine Measurement by ELISA

Concentrations of TGF-*β*1 levels in serum and the cell supernatant were measured using ELISA kits (Abcam). All procedures were performed in accordance with the manufacturer's instructions.

### 2.7. Western Blot Analysis

The right inferior lobe lung and MRC5 fibroblasts were lysed with RIPA buffer supplemented with a protease inhibitor cocktail (100 *μ*L/mL, GenDEPOT, Katy, TX, USA). Equal quantities of protein were separated on 6–10% SDS-PAGE and transferred onto polyvinylidene fluoride membranes (Millipore, Billerica, MA, USA). The membranes were blocked with 5% skim milk for 2 h at 37°C and then incubated with primary antibodies (*α*-SMA, TGF-*β* receptor I (TGF*β*RI), p-Smad2, Smad2, p-Smad3, Smad3, tissue inhibitor of metalloproteinase 1 (TIMP-1), and matrix metalloproteinase 9 (MMP-9), collagen I, and GAPDH; Abcam) overnight at 4°C. After exposure to HRP-conjugated secondary antibody for 2 h at 37°C, the membranes were analyzed with ECL reagents (Millipore). Relative protein expression levels were determined by scanning densitometry (ChemiDoc XRS + Systems, Bio-Rad Laboratories, Hercules, CA, USA) and analyzed using Image Lab 5.0 software (Bio-Rad Laboratories).

### 2.8. Statistical Analysis

All results are expressed as means ± SD. All graphing and statistical analyses were performed using GraphPad Prism 5.0 software (GraphPad, La Jolla, CA, USA). Statistical significance between two groups over time was evaluated using two-way analysis of variance (ANOVA), and one-way ANOVA followed by Tukey's post hoc test was used for comparison of data among multiple groups.* p* values ≤ 0.05 were considered statistically significant.

## 3. Results

### 3.1. Ginsenoside Rg1 Ameliorates CS-Induced Emphysema

**Figure 1:**
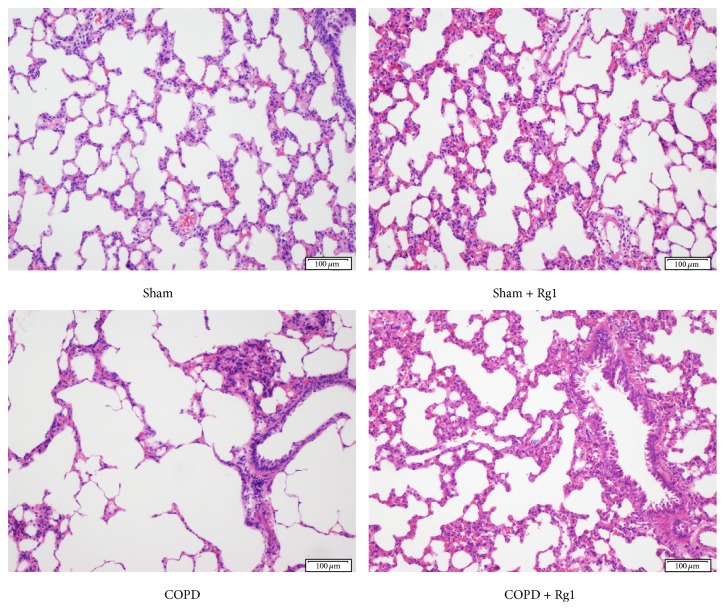
Effects of ginsenoside Rg1 on pulmonary histopathology in COPD rats. H&E staining of lung tissues. Scale bar = 100 *μ*m. All fields shown are representative of at least six fields observed in four rats for each group.

H&E staining was performed to evaluate pathological changes in lung tissues. After 12 weeks of CS exposure, the COPD group showed evident necrosis and shedding of bronchial mucosal epithelium, as well as marked inflammatory cell infiltration, in contrast to the Sham group. These changes were ameliorated by the administration of ginsenoside Rg1 (Figure 1). Moreover, MAN was significantly reduced, whereas MLI increased in the COPD group compared with the Sham group. The addition of ginsenoside Rg1 in the CS-exposed group markedly elevated MAN and decreased MLI, demonstrating that ginsenoside Rg1 suppressed airway disorganization. There was no significant difference between the Sham group and the Sham + Rg1 group (Table 1).

### 3.2. Ginsenoside Rg1 Attenuates CS-Induced Pulmonary Fibrosis

**Figure 2:**
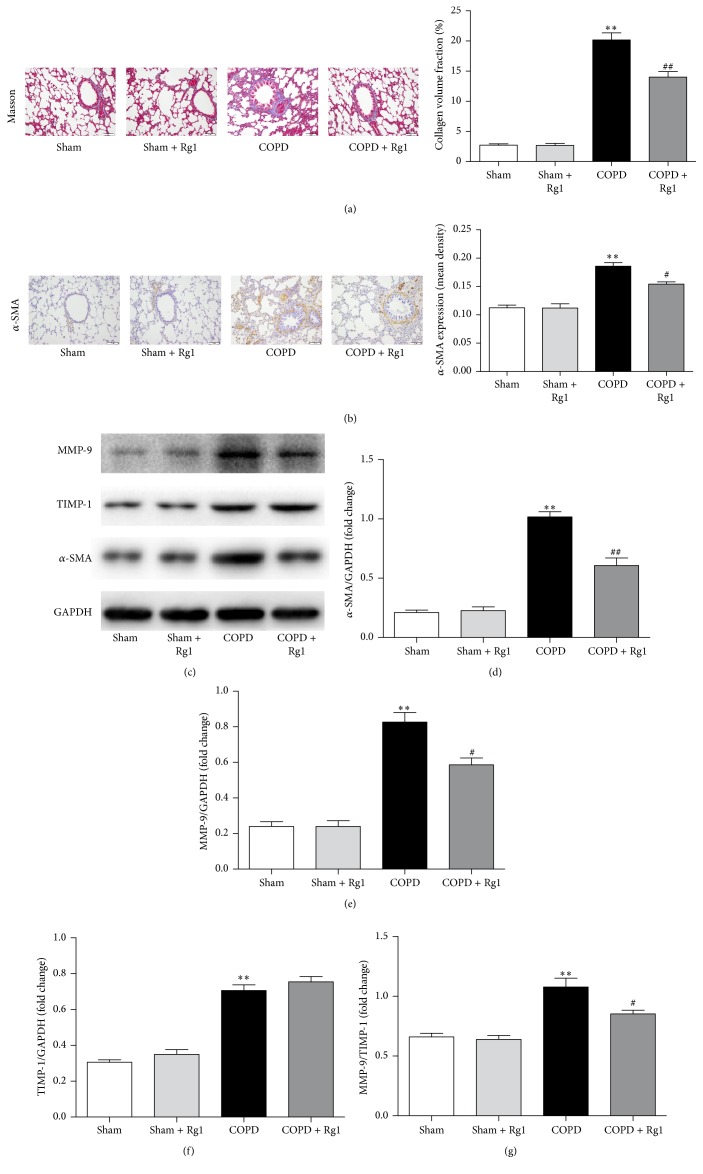
Effects of ginsenoside Rg1 on airway fibrosis in chronic obstructive pulmonary disease (COPD) rats. (a) Masson trichrome staining of lung tissues. Scale bar = 100 *μ*m. All fields shown are representative of at least six fields observed in four rats for each group. Quantitative collagen and elastin fiber assay was performed using Image-Pro Plus 6.0 software. (b) Immunohistochemical staining of *α*-SMA in lung tissues. Scale bar = 100 *μ*m. All fields shown are representative of at least six fields observed in four rats for each group. Quantification of *α*-SMA was carried out using Image-Pro Plus 6.0 software. (c) Protein expression of *α*-SMA, MMP-1, and TIMP-1 in lung tissues was determined by western blot analysis. (d–f) Quantitative analysis of *α*-SMA, MMP-9, and TIMP-1 protein expression. (g) Ratio of MMP-9 to TIMP-1. Data are shown as mean ± SD of at least three independent experiments, each performed in triplicate. ^*∗∗*^*p* < 0.01 versus the Sham group; ^#^*p* < 0.05 and ^##^*p* < 0.01 versus the COPD group.

Collagen and elastin fibers were visualized with Masson trichrome staining. The COPD group showed higher collagen and elastin signals than the Sham group; ginsenoside Rg1 treatment decreased collagen and elastin fibers (Figure 2(a)).

Pulmonary fibroblasts are a critical source of fibrotic matrix, and *α*-SMA is a marker of fibroblast activation. Immunohistochemical staining revealed that the expression of *α*-SMA significantly increased in the interstitium of the lung, peribronchial, and perivascular tissue of the COPD group in comparison with the Sham group, and the effect was suppressed by ginsenoside Rg1 administration (Figure 2(b)). Furthermore, western blot results confirmed upregulation of *α*-SMA protein levels in the COPD group and decreased *α*-SMA expression in the COPD + Rg1 group (Figures 2(c) and 2(d)).

Fibroblast activation contributes to tissue remodeling by secreting ECM molecules, including MMPs, TIMPs, and collagens. MMPs are a family of structurally related enzymes capable of degrading ECM. MMP-9 is involved in the pathogenesis of COPD airway remodeling [16] and is inhibited by TIMP-1 in a 1 : 1 proportion. We found that the COPD group had significantly upregulated MMP-9 and TIMP-1 levels in lung tissues. Moreover, the ratio of MMP-9 to TIMP-1 increased significantly in the COPD group compared with the Sham group. Notably, treatment with ginsenoside Rg1 effectively downregulated the expression of MMP-9 and the ratio of MMP-9 to TIMP-1 (Figures 2(c), 2(e), 2(f), and 2(g)).

### 3.3. Effects of CSE and Ginsenoside Rg1 on the Viability of MRC5 Fibroblasts

**Figure 3:**
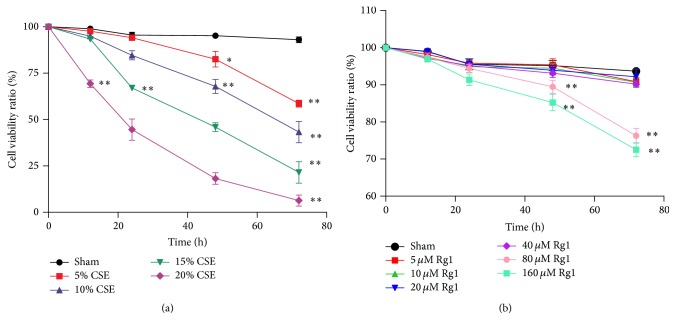
Effects of cigarette smoke extract (CSE) and ginsenoside Rg1 on the viability of pulmonary fibroblasts. (a) Cytotoxicity of CSE to MRC5 cells was measured using the CCK-8 assay. (b) Effect of ginsenoside Rg1 on cell viability. Values are expressed as the mean ± SD of three independent experiments, each performed in triplicate. ^*∗*^*p* < 0.05 and ^*∗∗*^*p* < 0.01 versus the corresponding Sham group.

We first examined the growth-inhibition effect of CSE on MRC5 fibroblasts using a CCK-8 assay. MRC5 cells were cultured in the presence of CSE (5%, 10%, 15%, and 20%) for 12, 24, 48, and 72 h. Differences in cell viability were not significant at 12 h except in the 20% CSE group; after 24 h, CSE markedly decreased cell viability in a dose- and time-dependent manner. The 48 h incubation displayed striking toxicity (>50% viability decrease) in the 15% and 20% CSE groups, while the 72 h incubation notably decreased cell viability in all CSE groups (>40% viability decrease) (Figure 3(a)). Therefore, MRC5 fibroblasts were treated with 10% CSE for 48 h in subsequent experiments. Next, we evaluated the effect of ginsenoside Rg1 on MRC5 cell viability. Cells were incubated with various concentrations of ginsenoside Rg1 (5 *μ*M, 10 *μ*M, 20 *μ*M, 40 *μ*M, 80 *μ*M, and 160 *μ*M) for 12, 24, 48, and 72 h. When cells were treated with 80 *μ*M and 160 *μ*M ginsenoside Rg1 for 48 h, cell viability decreased by 10% and 16%, respectively; cells treated for 72 h displayed a decrease of 14% and 21%, respectively, compared with cells in the Sham group (*p* < 0.01). Treatment with 5–40 *μ*M ginsenoside Rg1 showed only a minor effect on cell viability (Figure 3(b)). Thus, subsequent observations were performed following a 40 *μ*M ginsenoside Rg1 treatment.

### 3.4. Ginsenoside Rg1 Protects against CSE-Induced Lung Fibroblast Transdifferentiation

**Figure 4:**
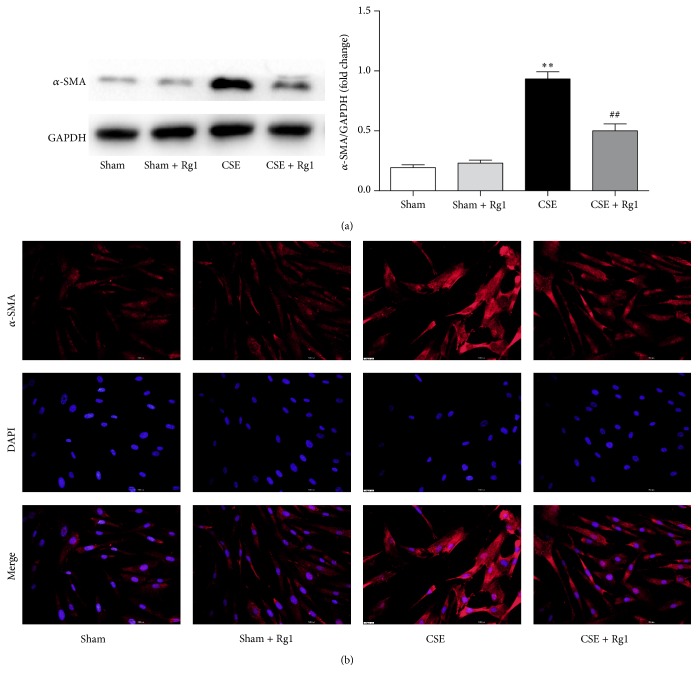
Ginsenoside Rg1 inhibited cigarette smoke extract- (CSE-) induced transdifferentiation of pulmonary fibroblasts. (a) Western blot and quantitative analysis of *α*-SMA protein expression. (b) Immunocytofluorescence assay of *α*-SMA (×200 magnification). Scale bar: 200 *μ*m. *α*-SMA is stained red; nuclei stained with DAPI are blue. Blots are representative of at least four independent experiments. All data are shown as mean ± SD of at least three independent experiments, each performed in triplicate. ^*∗∗*^*p* < 0.01 versus the Sham group; ^##^*p* < 0.01 versus the CSE group.

To investigate the effect of ginsenoside Rg1 on lung fibroblast transdifferentiation, MRC5 fibroblasts were pretreated with ginsenoside Rg1 (40 *μ*M) for 1 h and then exposed to 10% CSE for 48 h. Next, the expression of *α*-SMA, a marker of myofibroblasts, was evaluated by western blot and immunofluorescence staining. Western blot analysis showed that the protein expression of *α*-SMA increased significantly in response to CSE exposure and that this effect was suppressed by pretreatment with ginsenoside Rg1 (Figure 4(a)). Immunofluorescence staining indicated a similar effect of ginsenoside Rg1 on *α*-SMA expression (Figure 4(b)). These findings provide evidence that ginsenoside Rg1 may regulate transdifferentiation of lung fibroblasts.

### 3.5. Ginsenoside Rg1 Decreases ECM Deposition in Lung Fibroblasts Stimulated by CSE

**Figure 5:**
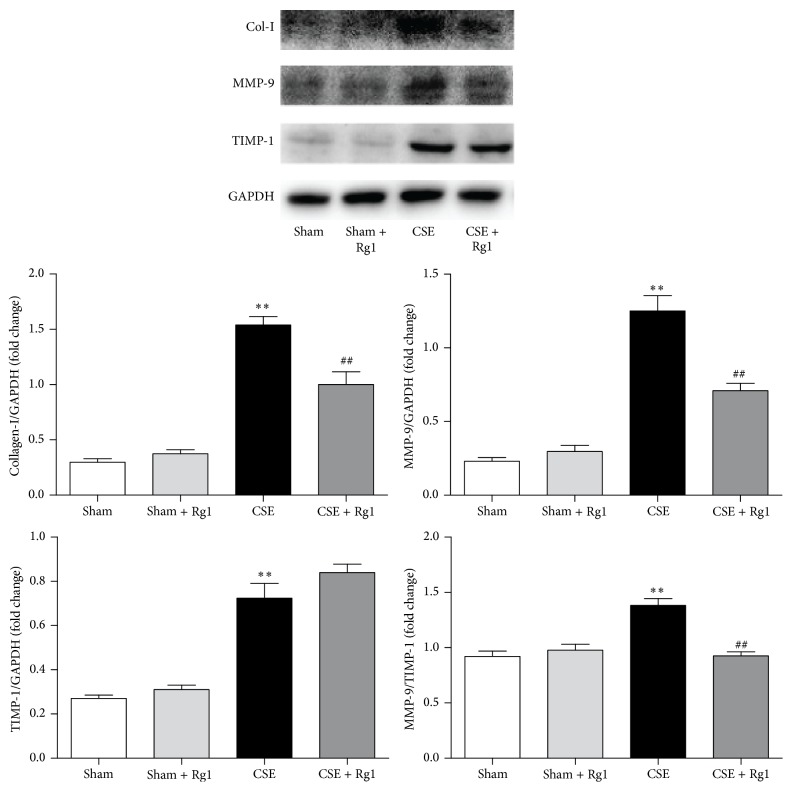
Ginsenoside Rg1 decreased cigarette smoke extract- (CSE-) induced extracellular matrix (ECM) deposition in pulmonary fibroblasts. Collagen I, MMP-9, and TIMP-1 protein levels were evaluated by western blot and quantitative analysis. Blots are representative of at least four independent experiments. All data are shown as mean ± SD of at least three independent experiments, each performed in triplicate. ^*∗∗*^*p* < 0.01 versus the Sham group; ^##^*p* < 0.01 versus the CSE group.

Compared with the Sham group, protein levels of collagen I, MMP-9, and TIMP-1 were significantly elevated in the CSE group (1.26-, 5.21-, 2.67-fold increase, respectively, *p* < 0.01 versus the Sham group). Furthermore, the ratio of MMP-9 to TIMP-1 was higher in the CSE group. Ginsenoside Rg1 administration attenuated increases in collagen I and MMP-9 and redressed the ratio of MMP-9 to TIMP1 (Figure 5).

### 3.6. Ginsenoside Rg1 Regulates the TGF-*β*1/Smad Signaling Pathway

**Figure 6:**
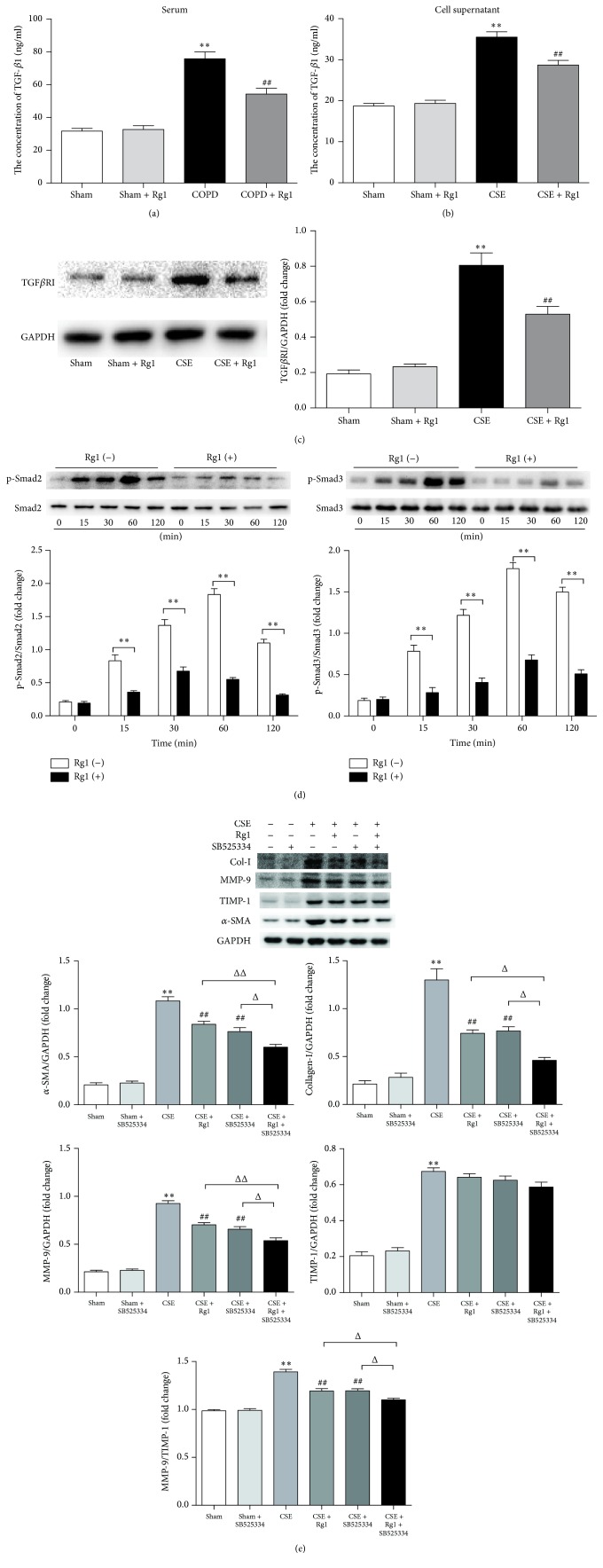
Ginsenoside Rg1 inhibited TGF-*β*1/Smad pathway in pulmonary fibroblasts stimulated by cigarette smoke extract (CSE) and mimicked the effect of SB525334 on CSE-induced pulmonary fibroblasts. (a) The concentration of TGF-*β*1 in serum was determined by ELISA. (b) The concentration of TGF-*β*1 in the cell supernatant was determined by ELISA. (c) Western blot and quantitative analysis of TGF*β*RI protein expression. (d) Western blot and quantitative analysis of p-Smad2 and p-Smad3 protein expression. (e) Western blot and quantitative analysis of *α*-SMA, collagen I, MMP-9, and TIMP-1 protein expression. Data (a, b, c, and e) are shown as the mean ± SD. ^*∗∗*^*p* < 0.01 versus the Sham group; ^##^*p* < 0.01 versus the CSE group; ^Δ^*p* < 0.05 and ^ΔΔ^*p* < 0.01 versus the CSE + Rg1 + SB525334 group. Data (d) are expressed as the mean ± SD. ^*∗∗*^*p* < 0.01 versus the corresponding ginsenoside Rg1(−) group.

Previous findings have indicated that the TGF-*β*1/Smad signaling pathway is closely associated with the genesis and development of pulmonary fibrosis [17]. TGF-*β*1 is a key switch in the process of fibrosis. To examine the molecular mechanism of ginsenoside Rg1 in CS-induced fibroblast transdifferentiation, we first evaluated the expression levels of TGF-*β*1 in the serum and cell supernatant using ELISA. Our findings indicated that CS or CSE exposure significantly induced TGF-*β*1 production, which was effectively reduced by ginsenoside Rg1 treatment both in vivo and in vitro (Figures 6(a) and 6(b)). Next, we tested TGF*β*RI expression in MRC5 cells. Western blot analysis showed that CSE-induced MRC5 cells exhibited decreased levels of TGF*β*RI following ginsenoside Rg1 treatment (Figure 6(c)).

We subsequently tested whether Smad2 and Smad3 were regulated by ginsenoside Rg1. Western blot analysis demonstrated that Smad2 and Smad3 were phosphorylated within 15 min following CSE stimulation, and the phosphorylations were highest 1 h after CSE stimulation. Compared with the CSE group, the p-Smad2/Smad2 and p-Smad3/Smad3 ratios in the CSE + Rg1 group were much lower, indicating that CSE-induced Smad2/3 activation was suppressed by ginsenoside Rg1 pretreatment (Figure 6(d)).

To confirm the role of TGF-*β*1/Smad signaling in inducing lung fibroblast transdifferentiation and ECM deposition, we determined the effect of the specific TGF*β*RI inhibitor SB525334, a selective inhibitor of activin receptor-like kinase 5 which can block the phosphorylation and nuclear translocation of Smad2/3 [18], on CSE-induced MRC5 fibroblasts. *α*-SMA, collagen I, and MMP-9 protein expression and the ratio of MMP-9 to TIMP-1 were downregulated by 29.5%, 41.1%, 29.1%, and 14.2%, respectively, in CSE-stimulated MRC5 cells treated with SB525334; there were no statistically significant differences between these groups and the CSE + Rg1 group. Furthermore, the reduction of *α*-SMA, collagen I, and MMP-9 and the ratio of MMP-9 to TIMP-1 induced by ginsenoside Rg1 were amplified by adding SB525334 (44.6%, 64.6%, 42.9%, and 20.9%, respectively) (Figure 6(e)).

## 4. Discussion

COPD is a dynamic process encompassing chronic lung injury and airway remodeling. Increasing resistance in peripheral airways less than 2-3 mm in diameter is one of the main pathological progressions of COPD and is primarily caused by small airway fibrosis [19, 20]. The characteristic features include fibroblast/myofibroblast transdifferentiation and accumulation of ECM components in the lung. The present study demonstrated the protective effect of ginsenoside Rg1 against CS-induced chronic lung injury/fibrosis in rats and pulmonary fibroblasts.

Activation of fibroblasts and conversion into myofibroblasts play a crucial role in many fibrotic diseases. The activated fibroblasts contribute to the deposition of collagen and other ECM components [21, 22], which increase the thickness of small airways and contribute to airflow limitation, leading to pulmonary fibrosis and airway remodeling. CS is a key risk factor in COPD and has been reported to promote the transition of fibroblasts to myofibroblasts [4]. In the present study, we observed by immunohistochemistry and western blotting that COPD rats displayed high expression of *α*-SMA, which is a hallmark of myofibroblasts, in lung tissues, whereas the variations could be attenuated by ginsenoside Rg1 treatment. At the cellular level, CSE stimulation increased *α*-SMA protein expression in MRC5 fibroblasts, while ginsenoside Rg1 treatment decreased *α*-SMA expression significantly. These results indicate that ginsenoside Rg1 may inhibit transactivation of pulmonary fibroblasts.

One of the most precise pathological characteristics of airway fibrosis is the excessive accumulation of ECM, mainly proteoglycan, elastin, collagen, and fibronectin, resulting in distinctive structural disorganization of the lung, which is associated with an imbalance in the ratio of MMPs to TIMPs [23]. Several studies have demonstrated that MMP-9 protein and mRNA levels are higher in the lung tissue and induced sputum of COPD patients [24, 25]. Serum TIMP-1 concentrations have been found to increase significantly in COPD patients [26, 27]. However, Yao et al. [28] found markedly low levels of TIMP-1 in COPD patients. In the current study, we observed that CS exposure enhanced MMP-9 and TIMP-1 protein levels, as well as the ratio of MMP-9 to TIMP-1, both in vivo and in vitro. Therefore, an excess of MMP-9, compared with TIMP-1, may result in excess ECM degradation, leading to airway remodeling. Moreover, our data showed that collagen I expression was higher in the CSE group than in the Sham group, which was similar to the effect observed in COPD rats. The overproduction of collagen may be due to the enhanced transdifferentiation of pulmonary fibroblasts to the myofibroblast phenotype, leading to excessive secretion of ECM proteins. In addition, we noted that ginsenoside Rg1 reduced the degree of airway fibrosis through pathological manifestation, downregulated the expression of MMP-9 and collagen I, and balanced the ratio of MMP-9 to TIMP-1, indicating that ginsenoside Rg1 suppressed the process of pulmonary fibroblast transdifferentiation and ECM deposition.

The TGF-*β*1 signaling pathway is a crucial driver of fibroblast activation, excess ECM accumulation, and pathogenesis of pulmonary fibrosis [29–31]. The downstream effects of TGF-*β*1 are mediated by Smads, a family of intracellular transcription factors [32, 33]. Ahn et al. found that an extract of* P. ginseng* inhibited TGF-*β*1-induced fibrosis by suppressing the phosphorylation of Smad2 and Smad3 [34]. This suggests that ginsenoside Rg1 may suppress fibroblast/myofibroblast transdifferentiation via the TGF-*β*1/Smad signaling pathway. Consistent with the in vivo finding, the production of TGF-*β*1 was highly elevated in CSE-stimulated pulmonary fibroblasts. Moreover, the protein expression of TGF*β*RI, p-Smad2, and p-Smad3 was increased in the CSE group, which was associated with increased synthesis of ECM, suggesting an autocrine loop in maintaining the fibrotic reaction. However, with ginsenoside Rg1 treatment in CSE-induced fibroblasts, the TGF-*β*1/TGF-*β*R1/Smad2/3 interactions in airway fibrosis were notably inhibited. Ginsenoside Rg1 significantly reduced the expression of TGF-*β*1, TGF*β*RI, p-Smad2, and p-Smad3. Furthermore, ginsenoside Rg1 mimicked the effect of SB525334, a selective inhibitor of TGF*β*RI capable of alleviating the phosphorylation and nuclear translocation of Smad2/3, on reduction of *α*-SMA, collagen I, and MMP-9 levels, as well as on restoration of the MMP-9 to TIMP-1 ratio. Although SB525334 amplified the suppression of fibroblast/myofibroblast transdifferentiation and ECM deposition in the presence of ginsenoside Rg1, the inhibitory effects were inferior to the superposed suppression of ginsenoside Rg1 and SB525334, indicating an overlapping therapeutic area between ginsenoside Rg1 and the TGF*β*RI inhibitor. These data suggest that ginsenoside Rg1 attenuates CSE-induced fibroblast/myofibroblasts transdifferentiation and ECM deposition, at least in part, through the TGF-*β*1/Smad signaling pathway.

It should be noted that apart from the Smad pathway, TGF-*β*-related protein also activates other signaling pathways, such as MEK/ERK, Wnt/*β*-catenin, and PI3K/Akt [35, 36]. Further investigations into the relationships between ginsenoside Rg1 and Smad-independent pathways are warranted. Additionally, a detailed analysis of TGF*β*RI should be performed to elucidate the precise molecular mechanisms of ginsenoside Rg1, which may provide an attractive therapeutic target for small airway fibrosis in COPD.

## 5. Conclusion

The present study demonstrates that ginsenoside Rg1 can act as a potent intervention against airway fibrosis in COPD. The blockade of the TGF-*β*1/Smad pathway may be an underlying mechanism by which ginsenoside Rg1 protects against chronic lung disease associated with fibrosis.

## Figures and Tables

**Table 1:** Comparison of mean alveoli number (MAN) and mean linear intercept (MLI) in rats of the four groups.

Group	*N*	MAN (×10^6^/m^2^)	MLI (×10^−6^/m)
Sham	10	356.7 ± 20.57	46.68 ± 3.23
Sham + Rg1	10	345.9 ± 17.44	48.17 ± 2.51
COPD	10	172.8 ± 18.78^*∗∗*^	78.95 ± 4.69^*∗∗*^
COPD + Rg1	10	252.5 ± 16.77^##^	64.39 ± 3.97^##^

^*∗∗*^ *p* < 0.01 versus the Sham group; ^##^*p* < 0.01 versus the COPD group.
